# Round the Hospitals

**Published:** 1917-11-24

**Authors:** 


### ROUND THE HOSPITALS.

Their Majesties the King and Queen must have had an unusually interesting afternoon on the 17th instant, when they paid a visit to the Whipps Cross War Hospital, Walthamstow, attended by Lord Stamfordham, Commander Sir Charles Cust, Bart., and Countess Fortescue. Their Majesties were received by Mr. Hayes Fisher, President of the Local Government Board, who presented Mr. C. H. Ward, chairman of the Guardians, and Mrs. Ward. Miss L. G. Ward, their daughter, presented a bouquet to the Queen, the flowers in which were distributed to the patients in the wards by Countess Fortescue at the Queen's request. His Majesty, through the personal service he has rendered to the sick by his visits to hospitals, in accordance with his deliberate plan which he has pursued for many years, has acquired considerable practical knowledge, and has become a most efficient hospital visitor. Their Majesties have the talent of organisation well developed, and when they found that it occupied a considerable time to together inspect the wards and talk freely to the patients, they divided forces. The King questioned the soldier patients as to the nature of their injuries, the length of time each had been in the ward, and where his home was. Many Australians and Canadians were in the hospital, in addition to English soldiers. In one ward two of the inmates, Lance-Corporal C. Mounsey, of the Yorkshire Regiment, and Rifleman F. Simmons, of the Rifle Brigade, had won the Military Medal, and His Majesty took the opportunity to bestow these decorations in person, to the no small delight of Mrs. Simmons, who was visiting the hospital and seated by her husband's bed. The Queen and Princess Mary devoted their attention chiefly to the women, and the kindly interest and graciousness of the Royal visitors gave great pleasure and excited much appreciation amongst the patients.

The King is at his best when rendering personal service, and the sympathy he excites was clearly shown in the conversation he had with his soldiers in the wards. In reply to a question, one soldier explained that his wound was inflicted by a bayonet. When His Majesty asked him if he had had a chance of doing a bit of bayoneting in return, the soldier laconically answered, "Some." From Walthamstow Their Majesties proceeded to Forest House, a convalescent home, and, meeting a convoy of ambulances drawn up in line, as well as a guard of honour supplied by the boys of the Naval Brigade, His Majesty heard that one of the ambulance drivers was a woman who had the reputation of being a diligent and clever driver. In reply to an inquiry where she had gained her skill for the work, the King found that she had had seven years' experience, and had formerly driven her own car. According to the *Times*, His Majesty's comment was: "Jolly good of you to give your time and talents to a work of mercy like this." While Their Majesties were inspecting Forest House, a small boy, Harold MacDermott, about eight years of age, had been placed in a bed in the ward with a bandaged head. The lad, however, was so anxious to see the King and Queen that, hoping to escape notice, he took off his bandages, and with only his nightshirt on ran to an open space past which Their Majesties were to walk. He was caught by a nurse, who wrapped him in a shawl, readjusted his bandages, and held him in her arms. The account of the boy's exploit amused the Queen very much, and the King expressed the hope to the nurse that the little lad would not catch a cold or suffer any hurt from his keenness.

The visit of the King and Queen to Whipps Cross War Hospital is likely to be memorable in the history of British nursing. We have already referred to the King's action in presenting two Military Medals to patients entitled to them. At the Nurses' Home the Queen for the first time presented certificates to the nurses who had successfully passed their examination after three years' training. Her Majesty also presented the medals won by the first three successful candidates. It is a matter of no small importance that Queen Mary should have thus directly identified herself with the working nurse, by whom the patients throughout our voluntary system are cared for with commendable efficiency and much-appreciated kindness. Coming events cast their shadows before, and every British nurse will welcome and remember the incidents at Whipps Cross War Hospital on November 17, 1917, and hope that in the near future Her Majesty may become much more closely and nearly associated with British certificated nurses, their training, education, and welfare. It is a great honour to Poor-Law nurses that Queen Mary should have selected some of their body to be the recipients of the first medals and certificates of the kind presented by Her Majesty. They were won after three years' training at a Poor-Law nurse-training school, whose able matron, Miss L. Clark, has had the enterprise and wisdom to appoint a highly competent sister-tutor as one of her staff.

Dame Katherine Furse has resigned her position as Commandant-in-Chief of the Women's V.A.D. organisation. Lady Ampthill, who did good service as wife of the Governor of Madras from 1899 to 1906, for which she received the Order of the Crown of India and the Gold Medal of the Kaisar-i-Hind, has taken up her duties as chairman of the Joint Women's V.A.D. Committee, and all communications should be addressed to her or to the Secretary of that Committee at Devonshire House, Picadilly, London, W. 1.

Lieut.-Gen. Sir Francis Lloyd, in command of the London District, speaking at the opening of an Auxiliary Hospital at Forest Hill, expressed the

**ROUND THE HOSPITALS—(*continued*).**

belief that many of the women of the V.A.D. were not sufficiently looked after. "We owe them," he declared, "the very best that we can give, and I am not at all certain that everywhere they are lodged and fed and made as comfortable as they ought to be. I say it advisedly, because I have seen some cases, not a thousand miles from here, where things are by no means as good as they should be."

SIR FRANCIS LLOYD shows commendable activity, and seems always ready to encourage everybody who takes pains to provide additional comfort and suitable occupation for our wounded soldiers, whereby the tedium of what Tommy calls "dead time" is annihilated. The Military Hospital, Endell Street, organised and managed by Dr. Garrett Anderson and Dr. Flora Murray, and staffed entirely by women, has deservedly won for itself golden opinions from all who are competent to appreciate and appraise its work. These ladies, with the aid of Lady Anderson, Mrs. Game, and Miss R. Wigram, have set themselves to encourage the soldier patients to engage in all sorts of fancy work, embroidery, and a multiplicity of handicrafts of various kinds, in the production of which the greatest interest is shown. This occupation has proceeded since February, and on the 15th instant an exhibition of wounded soldiers' work was opened by Sir Francis Lloyd at the Military Hospital, Endell Street, the object of the exhibition and sale being to defray the cost of the materials already used, which amounted to £90, and to purchase further materials, in order that the work may be continued. No more striking fact in evidence of the success of this excellent organisation could be given than that in eight months the men have worked 1,120 badges of their various regiments, belonging both to the Mother-country and the Dominions. The patients have been allowed to take away the badges with them as mementoes. We may add that what we have here recorded is but one of a multitude of instances which might be given of the united effort which is being made throughout the country to render the lot of the wounded sailors and soldiers as little irksome and as beneficial and uplifting as possible.

A CONFERENCE was held at the College of Ambulance, 3 Vere Street, W., on the 16th and 17th instant. On the first day Colonel Mayo Robson spoke on Surgical Developments during the War. In the course of his lecture he paid a high tribute to the work of the nursing service, and at its conclusion some most interesting demonstrations on the use of artificial limbs were given by two patients from Roehampton Hospital. Another lecture was given by Mr. A. Bagot upon Pediculosis and its control in connection with the spread of disease, and Mr. E. Hughes spoke on War Savings. On Saturday morning there were two papers, one on Developments in Public Health Work by Dr. Thomas, Assistant to the L.C.C. M.O., and the other on Health and Character Training in Children by Miss Norah March, B.Sc. In the afternoon a pleasant and much appreciated feature was the presence of Miss Thurston, Matron-in-Chief of the New Zealand Expeditionary Force. She was an excellent chairwoman, with a charming personality, who spoke on the enfranchisement of women in New York State, which had been granted without restrictions, and of the advantages which nurses had gained in New Zealand since State Registration was established there. The Hon. Albina Brodrick spoke at considerable length, and with her usual vigour and impulsiveness upon Professional Organisation and Development. The keynotes of her address were Democracy, Comradeship, and Organisation. We have not space to publish the address this week, but a full report of it will be found on page ii of *The Nursing Mirror* of November 24, 1917.

THE women of Reading, under the direction of the Mayoress, who is the chairman, and the Hon. Mrs. Broadbent, vice-chairman, of a special committee, are uniting in a splendid effort to increase the regular annual support by subscriptions given by the residents to the maintenance of the Royal Berkshire Hospital. A recent meeting testified to the willingness of the women residents to support their leaders in this commendable enterprise. The newly appointed treasurer, Dr. Gell, stated there were from three to four thousand private residents, of whom only about 190 were annual subscribers. He was confident there were many of the upwards of 3,500 non-subscribers who would readily become regular supporters of the hospital if they were made acquainted with the facts and were approached by the Ladies' Committee. The system pursued is to accept the division of the town into municipal wards, and to appoint a lady representative for each ward, whose work it will be to obtain the services of other ladies to help her, and so carry out a system of canvass everywhere. This plan has proved encouragingly successful in other places where it has been followed, under the leadership of representative women, and the response of Reading, led by the Mayoress and the Hon. Mrs. Broadbent, should, and we hope will result in a record addition to the hospital funds.

WE are glad to have continuous evidence of the wide interest which has been created by the publicity we have given to the amount of good work matrons and members of their staffs have been able to put into the cultivation of the garden and other vacant ground outside the hospitals and institutions where their main work lies. There seems to have been nearly everywhere a serious handicap caused by the impossibility of obtaining the necessary bottles for more than a limited quantity of fruit. Will every householder among our readers make a note of this, and give early notice to the matron of the hospital, with a garden, best known to them, that they will be able to send her by next June so many bottles and jars in which stores may have been delivered to them, for which many people have

**ROUND THE HOSPITALS—(*continued*).**

little or no use when empty. Another way would be to intimate their willingness to supply bottles and jars on request. We hope that every one of the readers of The Hospital will follow the example of Their Majesties the King and Queen and gladly render a little personal service to increase, or begin, their interest in the nearest hospital. Experience teaches such a course will bring them happiness, whilst it renders material service to the matrons and other willing workers who have shown so much enterprise under war conditions in saving expense by cultivating the land within their reach, and adding at the same time materially to the comfort of the patients and their staff. The Harvest Festivals this year have yielded much fruit and other commodities for hospital households this season.

As an instance of what hospital gardening can supply, we give the following account of the work during the late season of the matron of a fever sanatorium with eighty beds. Thirty pounds of jam and jelly were made; five dozen bottles, the maximum number obtainable, of fruit were preserved; a good supply of walnuts, damsons, onions, tomatoes, red cabbage, and beetroot was pickled; and in addition 50 lb. of apple and tomato chutney were made. The flower-beds, too, were utilised for the production of lettuces, radishes, onions, parsnips, carrots, turnips, etc., whilst all other ground available was dug up and planted with potatoes, artichokes, dwarf beans, cauliflowers, and many other vegetables, the seeds of which were sown by the matron, who afterwards planted out the seedlings in the allotted space with the happiest results. It is interesting to note that, although this was a first attempt on newly dug ground, the potatoes yielded an average crop. It is encouraging, too, that these successes have emboldened some to make their first experiments with poultry, the details and results of which are to be sent in due course for the information of readers of The Hospital.

"V.A.D.," in a letter to the *Morning Post* of the 14th instant, calls Sir Arthur Yapp's attention to the hard and continuous work entailed upon nurses in public institutions, and insists that in the new scale of rations they should be included under the head of women on heavy industrial or agricultural work. We have not yet come across an institution where they have reduced the scale of nurses' diets to the vanishing-point, as "V.A.D." would seem to imply. It is true that during an air-raid the day nurses in a certain Poor-Law institution, who were kept on duty from 6 o'clock one morning to 4 a.m. the next, were not supplied with any food, as the regulations enacted that no food, not even a cup of tea or a slice of bread-and-butter, should be given after 5 p.m. The Guardians on learning the facts voted an allowance of 4s. a week to all the officers, to enable them to buy extra food for themselves. We doubt this allowance being wise or sufficient. Indeed, we mention the case in the hope that the managers of each institution and hospital throughout the country will make it their business to go into this point and deal with it from a reasonable point of view. This is Sir Arthur Yapp's advice, and would, no doubt, be insisted upon if he had to deal with the matter directly.

What is wrong with Glasgow, a city which has often displayed exemplary liberality in the cause of charity, and especially of hospitals? We commend to the City Fathers and inhabitants of Glasgow the following, which we extract from a letter signed "Fair Play" which appeared in the *Glasgow Herald* of the 13th instant. "Fair Play" states "the nurse's working day in the city hospitals at Glasgow consists of ten hours. Off-duty time is two hours from the time of leaving the ward till the nurse enters it again in uniform. In these two hours she is expected to go out for the benefit of her health, but at least half an hour is consumed in getting to and from her ward and the changing of her uniform. Where is the time for much-needed rest? The remuneration, as everyone knows, is infinitesimal. The life is one of constant drudgery, demanding the utmost of her physical strength. When a breakdown occurs she is told her profession is to nurse, not to be nursed, and is made to feel generally that she can be dispensed with." "Fair Play's" is a grievous indictment, if true. Is it true in substance and in fact? We hope the editor of the *Glasgow Herald* will bring his correspondent to realise that it is clearly her plain duty to give the names of the hospitals where "Fair Play" maintains this state of affairs exists in the city of Glasgow.

In a war hospital a poor little specimen of humanity lay wounded nearly to death. No home, no father or mother could he call his own, and, living mainly by his wits, he had supported himself entirely from the age of twelve. He was pale and shrivelled, with the expression of a sick monkey, the lasting token of years of half-starvation. Yet in this frail mockery of a human frame there lived a courage that would grace a king. Joining the Army before attaining military age, he was but eighteen when a shell shattered both his legs, rendering necessary a flush amputation through each thigh. A day or two later, while he still was near to death, the ward sister, to cheer him up, said, "You know there's nothing the matter with you; you're only wounded." "Well," was his reply, "if there's nothing the matter with me, sister, it's time I got out of bed and looked for the other half."

☛ It is our invariable practice to pay for all items of news which we may publish. The minimum payment is 5s. Paragraphs of about twenty lines in length—that is to say, of 150 words or less—are preferred. Contributions should be addressed to The Editor, The Hospital, 28 and 29 Southampton Street, Strand, London, W.C. 2., and, to be in time for the current week's issue, should reach him by the first post on Mondays.

For Nursing Affairs in Ireland, see p. 166; Matrons' and Sisters' Appointments, see p. iv.

